# A feasibility study prior to an international multicentre paediatric study to assess pharmacokinetic/pharmacodynamic sampling and sample preparation procedures, logistics and bioanalysis

**DOI:** 10.1016/j.conctc.2018.08.008

**Published:** 2018-08-21

**Authors:** Agnes Maria Ciplea, Stephanie Laeer, Bjoern Bengt Burckhardt

**Affiliations:** Institute of Clinical Pharmacy and Pharmacotherapy, Heinrich-Heine-University, Dusseldorf, Germany

**Keywords:** Clinical trial, Pilot, Training concept, Pharmacokinetic, Pharmacodynamic, Feasibility, ACE, Angiotensin-converting-enzyme, Cmax, maximum serum concentration, ELISA, Enzyme-linked immunosorbent assay, EMA, European Medicines Agency, EU, European Union, FDA, U.S. Food and Drug Administration, GCP, Good Clinical Practice, LC-MS/MS, Liquid chromatography-tandem mass spectrometry, LENA, Labeling of Enalapril from Neonates up to Adolescents, PD, Pharmacodynamic(s), PK, Pharmacokinetic(s), pp, Percentage points, RAA system, Renin-angiotensin-aldosterone system, RIA, Radioimmunoassay

## Abstract

**Background:**

Variability in pre-analytical procedures such as blood sampling, sample preparation and transport can substantially influence bioanalytical results and subsequently impair reliability of data gathered during clinical trials. Especially in vulnerable populations, all efforts should be made to facilitate high-quality data extraction excluding unnecessary or repeated intervention.

**Methods:**

The EU-funded LENA project (Labeling of Enalapril from Neonates up to Adolescents) included a feasibility study in its preparatory procedures prior to first-in-child studies. Derived from a regular study visit, it encompassed all procedures, from sampling of two study-specific drugs and four sensitive humoral parameters to bioanalysis, to evaluate the quality of obtained samples and applicability of logistical and bioanalytical procedures. Drug administration to healthy adults was circumvented by pre-spiking the blood collection tubes with a drug solution. Five clinical sites were evaluated.

**Results:**

Clinical teams' preparedness and applicability of required sampling procedures was investigated in 18 volunteers, on-site. 97% of collected pharmacokinetic (PK) samples and 93% of samples for humoral parameters were obtained eligibly. Results met expectations, though one team had to be re-trained and performed a re-run. Planned procedures for sampling, sample preparation, transport and analysis were found to be suitable for being applied within paediatric trials.

**Conclusion:**

The concept of the presented feasibility study that simultaneously assesses PK/PD sampling, sample preparation, logistics and bioanalysis proved to be a promising tool for trial preparation. It revealed improperly installed processes and bottlenecks that required adjustments prior to start of recruitment. It facilitated high-quality conduct from the first moment of paediatric pivotal studies.

## 1 Introduction

The quality of pre-analytical procedures such as blood sampling as well as sample preparation and transport can considerably affect the results of bioanalytical determinations. This has been reported for electrolytes [1], metabolomics [2,3], protein markers [4] and drugs [[5], [6], [7]]. Especially in clinical trials, study outcomes might be biased without adequately controlled quality of the pre-analytical procedures [2,8], which cannot be compensated by highly sophisticated and validated methods of determination [2]. Thus, pre-analytical procedures ought to be standardised in advance, especially for trials in vulnerable populations, to prevent unnecessary or repeated interventions. This is typically done only through a short training of staff during the site initiation visit for a clinical trial.

Between 19% and 40% of randomised controlled paediatric trials were found to have been discontinued [9,10]. Amongst the main reasons for discontinuation were conduct problems including logistical and technical issues. Investigators have made some attempts to avoid trial failure due to non-adherence to protocol or incomplete data sets [11]. Small-scale studies such as pilot or feasibility studies prior to a main study are performed to verify the site performance and enhance trial success [12,13]. Some researchers even claimed that it seems unethical to conduct a study whose feasibility has not been verified [12]. Most of these feasibility or pilot studies focus on aspects of processes, resources, management and/or science of the main study [12]. For example, they may assess the randomisation procedure, recruitment rate or suitability of assessment procedures and outcome measures [11,13]. Ideally, they should target identified risk factors for successful completion of the main trial [14].

Especially prior to paediatric trials, pilot or feasibility studies should be performed to verify applicability of procedures and performance of clinical sites, to avoid unnecessary clinical investigations in the highly vulnerable paediatric population [15]. Therefore, investigators of the LENA trials ('Labeling of Enalapril from Neonates up to Adolescents') trials performed a feasibility study. They identified the most challenging aspects of trial conduct. These were the highly sophisticated blood-sample preparation procedures for pharmacokinetic (PK) and several pharmacodynamic (PD) parameters, aimed at avoiding incorrect determination of temperature-sensitive substances with short half-lives that subsequently causes non-reliable data.

## 2 Materials and methods

### 2.1 The LENA project

The LENA project is an international academic research project funded by the European Commission. It aims to investigate orodispersible enalapril minitablets administered to children suffering from heart failure. Alongside pharmacokinetics (PK), several sensitive humoral parameters for evaluation of pharmacodynamics (PD) are determined, aiming to improve the understanding of the maturating renin-angiotensin-aldosterone system (RAA system) and its response to drug therapy with angiotensin-converting-enzyme (ACE) inhibitors. The demanding sampling and sample preparation procedures have already been subject to joint, comprehensive training for involved clinical teams, prior to the presented feasibility study.

### 2.2 Feasibility study within the LENA project

The intended pivotal paediatric studies of the LENA project aim to obtain reliable pharmacokinetic data of the ACE inhibitor enalapril and its active metabolite enalaprilat (primary study endpoint) as well as to consistently determine alterations in humoral parameters (secondary study endpoint). To ensure that right from the beginning of the pivotal studies high quality PK and PD data is generated, the objective of the here presented feasibility study, was to investigate the clinical teams' ability to perform the challenging sampling-related procedures and obtain samples within the pre-defined specifications. It assessed feasibility of planned procedures related to collection, transport and bioanalysis of PK/PD samples.

### 2.3 Ethical approvals

Clinical sites obtained ethical approval (or confirmation that an approval for this specific kind of study was not required) before participating in the feasibility study (EK 1690/2015 at the Medical University of Vienna, WT/aj/247957 at the Erasmus Medical Center in Rotterdam, WAG/mb/15/037193 at the University Medical Center Utrecht, and REC no. 16/SC/0124 at the Great Ormond Street Hospital). In addition, the University Clinics' ethics committee in Dusseldorf, where the project's central laboratory is located, agreed on the protocol (Protocol No. 5118). The work has been carried out in accordance with the Declaration of Helsinki. The data protection concept/privacy rights of the investigation complied with the regulations of North Rhine-Westphalia (Germany) being regarded as one of the strictest in Europe.

### 2.4 Inclusion/exclusion criteria for participation in the feasibility study

Healthy adults without cardiovascular diseases and without any current medication (self-reported), aged between 18 and 50 years, were eligible for participation. Volunteers whose (self-reported) health condition raised medical concerns against blood withdrawal (e.g., anaemia) were excluded. All participants provided informed consent prior to the start of the study.

### 2.5 Study design and conduct

**Fig. 1:**
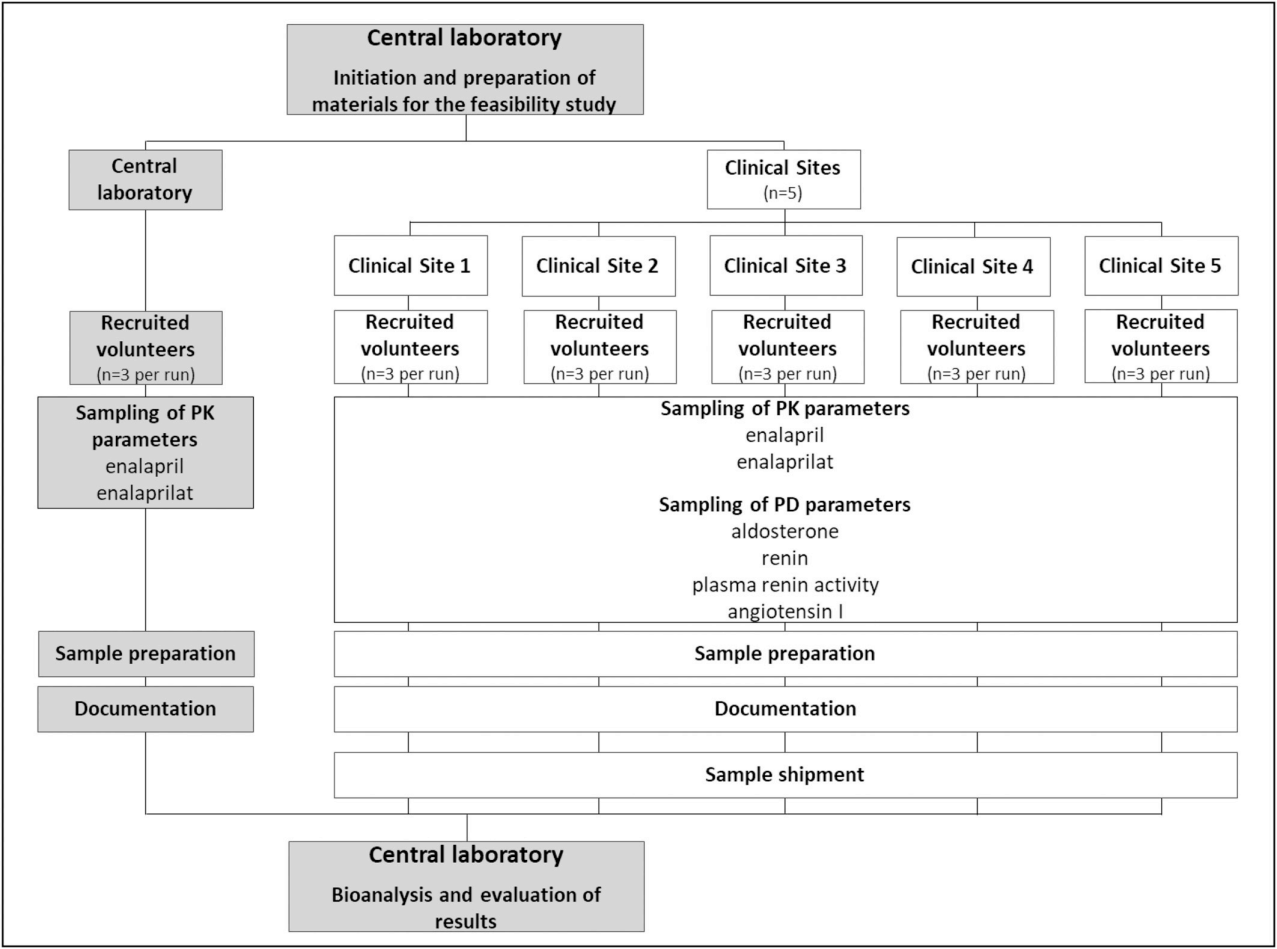
Flow chart of the intended study design of the feasibility study.

The feasibility study was designed to mimic the study sampling procedures of a regular LENA study visit within the paediatric studies. Details on performed procedures are provided in Fig. 1. The exact date of conduct was chosen individually for each clinical site, according to the anticipated start of recruitment. Areas assessed at the clinical sites included sampling, on-site sample preparation, documentation and dispatch of samples to the central laboratory of the LENA project for subsequent analysis as well as evaluation of results. Each clinical site performed corresponding procedures on three adult volunteers. Staff members were encouraged to rotate tasks, e.g., blood collection, documentation, and sample preparation. This ensured that every member of staff ran through all procedures that would come up during the trials. In this feasibility study, clinical teams of five clinical sites from four countries (Great Britain, The Netherlands, Austria, and Hungary) were involved.

Sampling requirements encompassed blood withdrawal for the compounds of interest in the paediatric trials of the LENA project. For the PK-related primary outcomes, the compounds are enalapril and its metabolite enalaprilat. The humoral parameters aldosterone, renin, plasma renin activity and angiotensin I are of interest as secondary outcome measures for exploratory PD investigations [[16], [17], [18]]. All samples were collected and labelled in accordance with the approved pseudonymisation process. Due to the sensitivity and poor stability of investigated peptides and hormones, all samples required immediate on-site sample preparation. Procedural instructions were applied as outlined in the corresponding LENA manual on sampling and sample preparation. This included a fixed sampling and sample preparation sequence, a strict time limit and specific temperature conditions to be applied during sampling, clotting time as well as centrifugation, depending on the type of the sample matrix. Blood samples for temperature sensitive humoral parameters were drawn with the blood collection tube enveloped in an ice pack and transported on ice to further preparation. All samples were centrifuged for 10 min at 2000 × *g*, at either room temperature or 4 °C depending on temperature sensitivity. The supernatant was subsequently transferred to cryo-tubes and snap frozen in a methanol/dry ice bath. Depending on the sensitivity of the substance of interest, applicable time limits to proceed from sampling to snap freezing of the supernatant were defined as 15 or 30 min. The consumables used and all on-site laboratory equipment utilised were identical to equipment intended for the paediatric trials, including the small-scale sampling material for paediatric use, the layout for labels and all form sheets required.

### 2.6 By-pass of drug administration

**Fig. 2:**
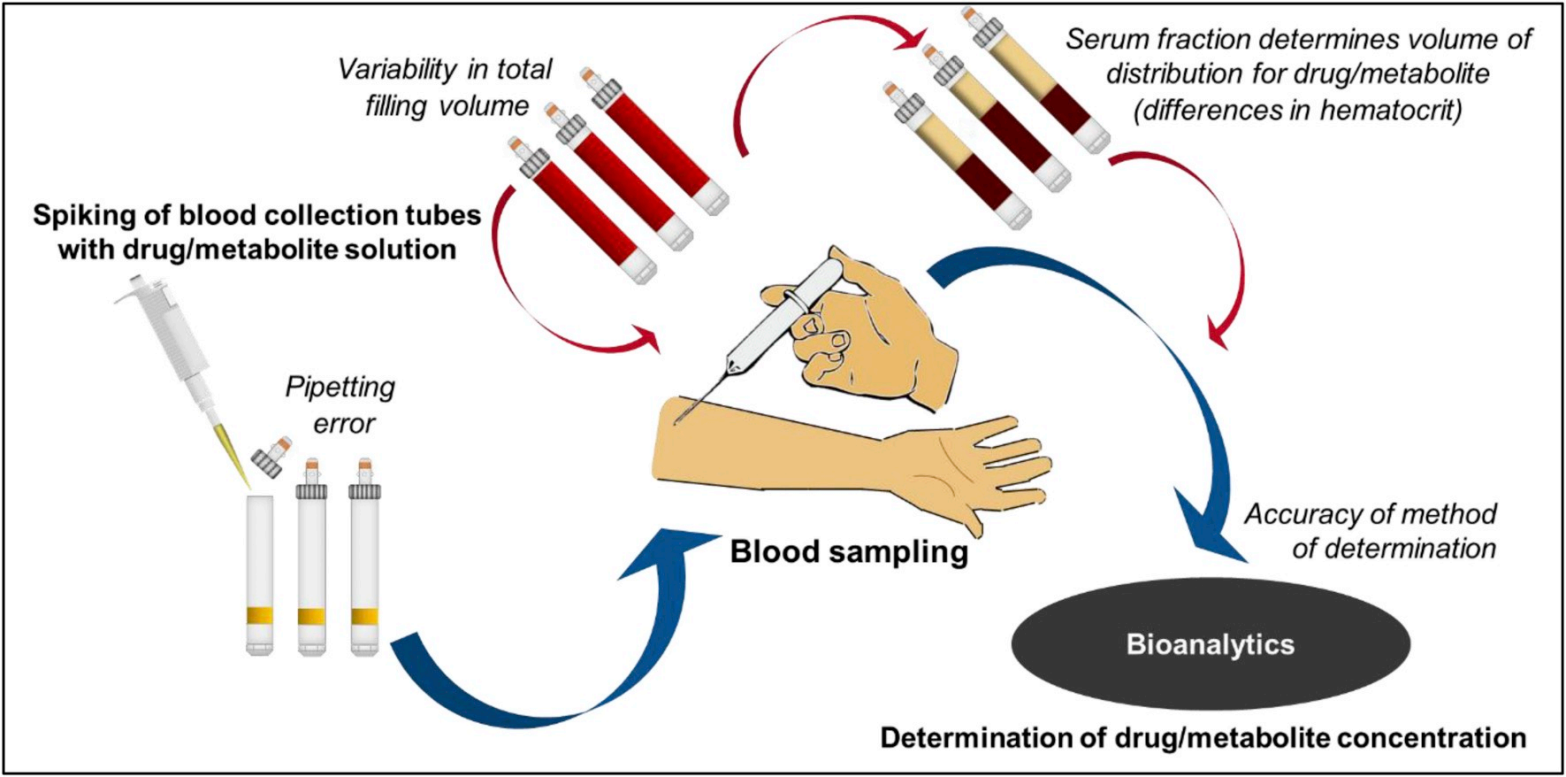
**Circumvention of drug administration in the feasibility study.** Procedures from spiking of blood collection tubes to the determination of drug concentration are shown by bold/blue arrows. Additional variability in results induced by this approach is indicated by thin/red arrows. (For interpretation of the references to colour in this figure legend, the reader is referred to the Web version of this article.)

Bearing in mind ethical constraints on unnecessary drug administration to healthy volunteers, a special concept to by-pass drug application was developed to avoid administration of a drug during the feasibility study. This avoidance was achieved by pre-spiking the blood collection tubes with a stock solution of the drugs. The spiking took place in batches at the Institute of Clinical Pharmacy and Pharmacotherapy (Dusseldorf, Germany). The spiking procedure is illustrated in Fig. 2. The volume and concentration of the spiked drug solution is based on calculations regarding the final concentrations per blood-filled tube without substantial dilution of blood collected. Targeted concentrations in whole blood were 50 ng/mL enalapril with 25 ng/mL enalaprilat and 12.5 ng/mL enalapril with 6.25 ng/mL enalaprilat. These concentration levels reflect expected Cmax of both compounds in a neonate/toddler as well as in an adolescent and were reached during the withdrawal of blood as drug solution and freshly collected blood mixed up. To avoid deviations in filling volume, thus affecting determined concentrations, discard tubes were provided to remove the air from the connective tubing before the first sample was drawn. Therefore, it was assumed that a comparable maximal filling volume could be obtained in all samples, if sampling was performed correctly.

### 2.7 Evaluation of bioanalytical results for PK samples (enalapril/enalaprilat)

#### 2.7.1 Acceptable range of results

To consider characteristics of the applied by-pass procedure for drug administration for the evaluation of results, setting specific factors had to be considered to establish an acceptable range of results. The range was based on following variables: First, based on applicable guidelines on bioanalytical method validation by the European Medicines Agency (EMA) [19] and the U.S. Food and Drug Administration (FDA) [20], acceptable accuracy was defined as a maximum deviation to the nominal/reference value of ±15%. Second, the pipetting error during pre-spiking and variability in the filling volume were also considered. The total pipetting error was compounded by a systematic and random error provided by the pipette manufacturer (Eppendorf, Hamburg, Germany) as 1.6% (model: Multipette® M4). Pre-examinations had indicated that the filling volume among the blood collection tubes differed by 1.1% if the vacuum approach is applied (Sarstedt, Nuembrecht, Germany). Third, a factor that had to be considered, due to its elementary influence within the concept of pre-spiked blood collection tubes, was the haematocrit. As both enalapril and enalaprilat have a blood to plasma ratio <1, the individual haematocrit affects the distribution volume of the drug and metabolite by defining the available serum fraction.

As no individual determination of haematocrit was performed in the volunteers, the lower reference value for females (35.8%) and the upper reference value for males (51.0%) were used to define the expected haematocrit range for the healthy volunteer population. Thereby, it was ensured that calculations account for volunteers with haematocrit values up to the margins of reference values for healthy adults. As no international standardised reference values are established, the reference values used were calculated as the mean of reference values applied in the participating clinical sites' routine laboratories. The 15.2% percentage points (pp) difference between 51.0% and 35.8% constitute a maximal variability of 29.8% between volunteers.

Based on these identified variables, the acceptance range was defined as 58%–153% of the mean reference concentration of enalapril and its active metabolite enalaprilat represented by Equations (2), (3).

(1) Limit (%) = 100 * AMVG * MHD * VBC * PE (%)

AMVG: acceptable maximal variation according to the EMA/FDA guidelines, MHD: maximum haematocrit difference in healthy adults, VBC: variability in the filling volume of blood collection tubes, PE: pipetting error.

Equation (1) *Formula for the calculation of acceptable ranges of accuracy for drug/metabolite results from pre-spiked blood sampling tubes compared to reference values.*

(2) Lower limit (%): 100 * 0.85 * 0.702 * 0.989 * 0.984 = 58%

(3) Upper limit (%): 100 * 1.15 * 1.298 * 1.011 * 1.016 = 153%

Equations (2), (3) *Calculated acceptance range of accuracy for the feasibility study, for drug/metabolite results from the pre-spiked blood sampling tubes compared to reference values.*

#### 2.7.2 Reference samples

Determined concentrations of each adult were compared to the mean concentration of three reference samples collected under ideal conditions at the central laboratory (Institute of Clinical Pharmacy and Pharmacotherapy, Heinrich-Heine University, Dusseldorf, Germany). This approach addresses possible distribution processes between plasma and blood cells as well as plasma protein binding and inevitable degradation processes during sample preparation. Blood collection tubes used to obtain these reference samples originated from the same batch of pre-spiked collection tubes used at the clinical site. The spiking procedure is illustrated in Fig. 2.

### 2.8 Evaluation of bioanalytical results for PD-related humoral parameters (aldosterone, renin, plasma renin activity, and angiotensin I)

Criteria to assess acceptability of results obtained for concentration/activity of the RAA system-related substances investigated were derived from established reference ranges for adults. Deviations from recommended sample preparation procedures can cause major degradation. Due to their sensitivity and poor stability, prompt, complete and correct on-site sample preparation was mandatory for high-quality and reliable sample results. Therefore, sample preparation had to take place immediately and be conducted by the clinical staff on the ward. Since these procedures exceed daily routine by far, it is prone to handling errors affecting the sample quality. Correct sampling and detectable values of concentration/activity in collected samples were defined as criteria for successful conduct. Detected values were expected to be above the lower limit of reference values in adults, as far as established. Since the feasibility study was implemented into daily routine at the ward, the sampling was performed in a sitting position without obligatory resting time. Owing to the missing resting time, reference values for standing position were used for evaluation. According to available reference values in adults, the concentration levels were expected to exceed the following limits: 22.1 pg/mL for aldosterone (MVZ Laboratory Dr. Limbach, Heidelberg, Germany), 1.68 pg/mL for renin (Spranger Laboratories, Ingolstadt, Germany) and 0.06 ng/(mL*h) for plasma renin activity (Laboratory Dr. Spranger, Ingolstadt, Germany). For angiotensin I, no lower reference value was provided by the analysing laboratory.

### 2.9 Evaluation of logistics

Since the courier services by DHL were contracted for the paediatric studies through the company's recently introduced clinical trial-specific service called “Medical Express”, the services and their reliability were assessed in the feasibility study. Due to limited access to dry ice at the clinical sites and with regard to the generally high workload on the wards, a care-free shipment approach was established by the central laboratory. Parcel pick-up was initiated remotely, and clinical sites were provided with parcels pre-filled with dry ice and required labels/documentation for sample transport. For both shipments together (outward and return), a total turnaround time of three days was scheduled. The timeliness, tracking function and reliability of the transport as well as the condition of the parcels (damaged/undamaged), functionality of provided temperature data loggers and general quality of the service were assessed.

### 2.10 Evaluation of laboratory procedures

Obtained samples were shipped on dry ice to the central laboratory (Bioanalytical Laboratory of the Institute of Clinical Pharmacy and Pharmacotherapy, Heinrich-Heine University, Germany). Determinations for enalapril, enalaprilat, aldosterone, renin, plasma renin activity and angiotensin I were performed at the institute or by an external partner (Spranger Laboratories, Ingolstadt, Germany). Drug concentrations of enalapril and its metabolite (enlaprilat) were determined using a low-volume LC/MS-MS method [21], whereas humoral parameters (aldosterone, renin, plasma renin activity and angiotensin I) were determined using enzyme-linked immunosorbent assays (ELISA) [22,23] or radioimmunoassays (RIA). The bioanalysis at the laboratories was performed blinded. The interface management of sample dispatch, sample registration and storage as well as bioanalysis were assessed in the feasibility study.

## 3 Results

Between November 2015 and March 2016, five clinical centres involved in the LENA project (83% of all clinical sites at this stage) performed the on-site feasibility study. The clinical sites were located in the Netherlands (2 sites), Austria (1 site), Hungary (1 site) and the United Kingdom (1 site). A sixth site in Serbia was not able to participate, due to national regulatory restrictions.

### 3.1 Study population

In total, 18 apparently healthy volunteers were recruited at the five clinical sites, whereof three were enrolled per site. Since one site repeated the feasibility study, three additional volunteers were recruited, thus resulting in a total of 18 participants.

To obtain reference samples, additional volunteers were recruited at the central laboratory. As the feasibility study was conducted successively in a site-by-site approach and subsequently lasted for about five month, it was necessary to recruit three times owing to stability reasons of pre-spiked blood collection tubes at the central laboratory. The latter results in a total of nine recruited volunteers at the central laboratory (3 runs with 3 volunteers each). Main characteristics of the volunteer population are presented in Table 1.

**Table 1:** Demographics of volunteers* Calculated, as volunteers provided only the year of birth. The presumed birthday is December 31 of a given year.

	Volunteers Clinical Sites (N = 18)	Volunteers Reference (N = 9)
Age* in years (±SD)	33 (±7)	29 (±3)
No. of females (%)	10 (55%)	5 (55%)

### 3.2 Results for the PK samples (enalapril and enalaprilat)

Using the pre-spiked blood collection tubes for evaluation of PK sampling performance by each site enabled determination of eligible concentration levels at all five clinical sites. The pre-spiking approach appeared to be suitable to evaluate the clinical team's ability to perform the PK sampling according to the intended trial procedures.

**Fig. 3:**
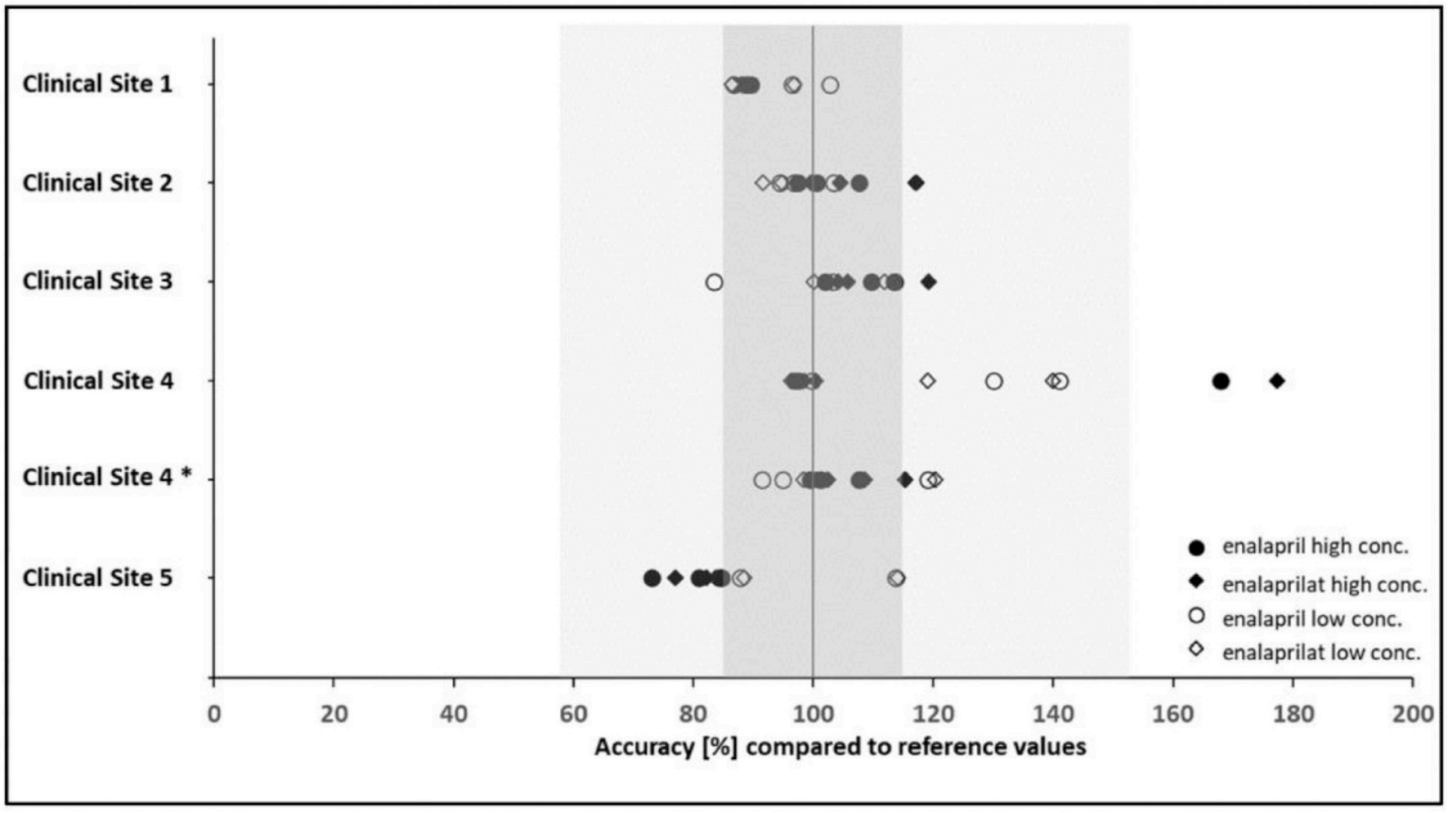
**Accuracy of enalapril/enalaprilat samples, obtained with pre-spiked blood collection tubes, in relation to reference values**. The dark grey area indicates the acceptable accuracy of the bioanalytical method according to EMA/FDA Guidelines (±15%). The light grey area indicates the extended tolerance range based on additional influencing factors of the artificial setting of the feasibility study (58%–153%). * denotes results from the re-run for the site that did not pass the feasibility study in its first attempt.

In total, 29 of 30 planned PK samples were collected during the first attempt of the feasibility study. Due to a technical handling error of the sampling material at clinical site 1, one PK sample with low concentration levels was lost. At four out of the five sites, results of both concentration levels and compounds were determined between 73% and 120% when compared to the reference values, hence within the defined acceptable accuracy range of 58%–153%. Obtained results for the two differently concentrated pre-spiked samples, displayed as accuracy (%) relative to the results of reference samples, are illustrated in Fig. 3.

In its initial performance, clinical site 4 exceeded the allowed deviation for one sample, where relative concentration values were determined as 168% for enalapril and 177% for enalaprilat. This result indicates too little blood was drawn within the respective tube to allow for appropriate dilution of the drug solution inside. In addition, this site had the highest variability of results, and its obtained accuracy levels varied between 96% and 177%. This was considered to be an unsatisfactory outcome, and it contributed to the decision to repeat the feasibility study at this site, which was completed successfully in its second attempt.

On overall analysis revealed some inter-site variability (e.g. site 2 vs site 5). This detected difference in accuracy was most probably caused by slight differences in the ward environment and equipment used. Since all sites performed finally according to the predefined specifications, no further investigation on the exact reasons concerning inter-site specific variability was conducted.

### 3.3 Results for the PD-related humoral parameters aldosterone, renin, plasma renin activity and angiotensin I

**Fig. 4:**
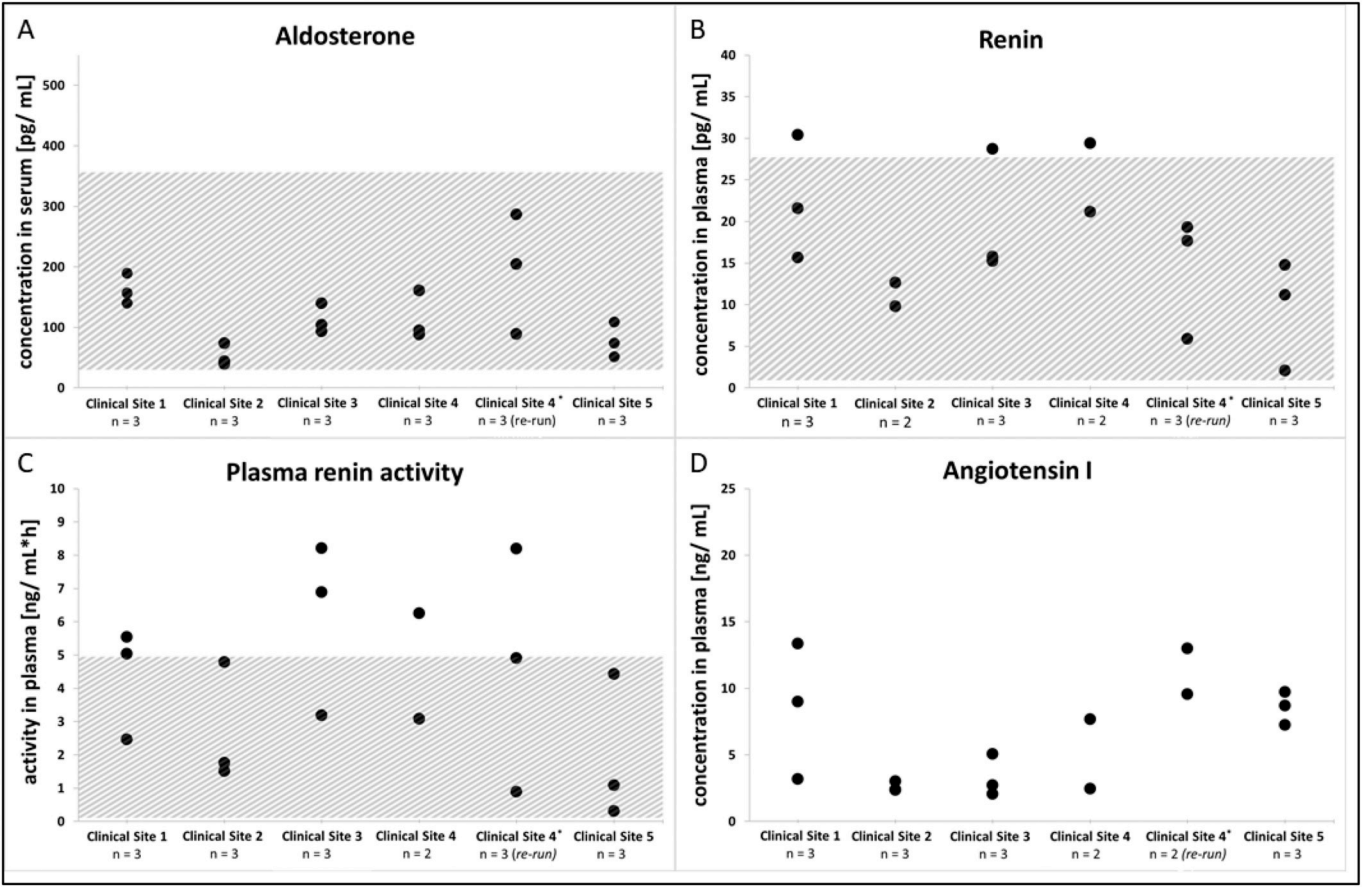
**(A**–**D) Results for humoral parameters aldosterone, renin, plasma renin activity and angiotensin I.** Each black circle represents an analytical result for an evaluable sample. Hatched areas indicate applied reference ranges for healthy adults, as far as established or given by the laboratory. * denotes analytical results from the re-run for sites that did not pass the feasibility study in their first attempt.

The developed on-site sample preparation procedures appeared appropriate and effective in collecting reliable results of humoral parameters at all sites. Unless no handling error during sample collection and on-site preparation occurred, detectable levels of all humoral parameters investigated were measured. Analytical results of samples eligible for analysis are illustrated in Fig. 4(A–D). Determined aldosterone levels were within the applied reference range of 22.1–353 pg/mL (standing position) for aldosterone (MVZ Laboratory Dr. Limbach, Heidelberg, Germany). Levels of plasma renin activity were found to be higher than the reference range of 0.06–4.96 ng/(mL*h) (Spranger Laboratories, Ingolstadt, Germany) in six different volunteers from four different clinical sites, with a maximal measured value of 8.2 ng/(mL*h). The same applies to renin, of which three samples from three different sites had values above the reference range of 1.68–27.66 pg/mL (Spranger Laboratories, Ingolstadt, Germany). The highest renin concentration determined was 30.42 pg/mL. Angiotensin I could be detected in all samples analysed. However, not all samples could be collected per volunteer. From a total of 60 pharmacodynamic samples that had to be drawn originally (four per volunteer), four samples had to be excluded from analysis. This was required because of either insufficient volume or handling errors resulting in unappropriate samples for bioanalysis. Three of these samples originated from clinical site 4. Analogue to the results for PK samples at clinical site 4, the accumulation of ineligible samples at this site contributed to the decision to re-train staff in PK plus PD sampling and perform a re-run of the study. In the re-run, one pharmacodynamic sample was missed due to a pipetting error.

The overall findings correspond to the author's expectation of results above the lower limit of reference values for adults, as apparently healthy volunteers were recruited.

### 3.4 Results for logistics

All parcels shipped by DHL “Medical Express” reached their destination intact, the day after dispatch, although not all were delivered within the announced time window. Information provided by the online tracking was accurate and available with an acceptably short lag time. Unfortunately, no automatic notifications were sent in case of delays in transport. Insulation and storage capacity proved to be sufficient to maintain dry ice for the duration of a three-day sample shipment cycle. Provided temperature data loggers were found to be unsuitable for usage on dry ice. The batteries drained too fast when exposed to these extremely low temperatures to enable any relevant data recording. Therefore, the purchase of suitable devices (testo® 184 t4) was initiated, to be used for shipments during the paediatric trials. Overall, services provided by DHL were assessed as appropriate to be used for the paediatric trials after calculation of cost-risk ratio.

### 3.5 Results for laboratory procedures

Recently implemented sample registration and storage documentation procedures, tailored to high sample throughput, allowed for fast and traceable sample handling at the central laboratory. Established workflows in accordance with GCP proved the bioanalytical teams' preparedness for timely sample preparation and measurements using ELISA and LC-MS/MS. Scheduled assay runs could be performed in time without protocol deviations, properly documented and reported.

## 4 Discussion

Our concept of a feasibility study that assesses on-site PK/PD sampling, sample preparation, logistics and bioanalysis simultaneously has proved to be a promising tool for trial preparation and is likely to have substantially contributed to the good quality of obtained samples during the main trials. Planned procedures and adequate training of clinical teams could be assessed by performing study-specific sampling for all PK/PD parameters of interest on healthy adult volunteers before investigations in the more vulnerable population of diseased children were initiated. Substances of interest were determined at expected levels, and planned procedures were found to be suitable for being applied within the paediatric trials.

Performing the current study to verify feasibility of planned procedures for the main trials, as recommended by researchers [12], had a substantial impact on the preparatory procedures. It became apparent that one site required re-training concerning study-related sampling and sample preparation procedures. It succeeded to obtain blood samples according to the pre-defined requirements only in its second attempt. The feasibility study appeared to be a capable tool of detecting sampling errors as too low volume and transfer of the whole blood instead of serum/plasma for analysis. It enabled retraining of clinical staff before study procedures were performed in children.

As recommended by researchers [24], the feasibility study focused on the complex PK/PD sampling procedures identified as a main risk for study failure for the planned trials. These complexity is a consequence of the sensitive substances investigated, e.g., angiotensin I; a PD parameter of the RAA system has an in-vitro half-life in non-anticoagulated whole blood of 3 min [25], and renin's precursor pro-renin is cryo-activated when exposed to temperatures between −5 °C and 4 °C [26]. Other investigators had focused in a similar manner but on other parameters of a planned study, such as feasibility and acceptability of randomisation; cost effectiveness, retention and suitability of outcome measures; as well as feasibility of a planned intervention. Thus, the current results demonstrate that highly sophisticated pre-analytical procedures, should be an objective of feasibility studies performed prior to a main trial.

The validity of the results of the current feasibility study is not limited by the study's conduct in healthy adults instead of in children, the population of the main trial. This was an ethically motivated decision, as it seemed inappropriate to perform invasive investigations in paediatric patients without benefit but only for testing purposes [15]. Furthermore, the investigated peptides/hormones aldosterone, renin, plasma renin activity and angiotensin I are inherent components of the RAA system. They are physiologically present in all human beings. The absolute levels may vary between healthy individuals and paediatric patients, but they are not decisive for the verification process, as investigating the impact of heart disease and/or age on RAA system-related substances was not an objective of the feasibility study. Therefore, the inclusion of healthy adults was adequate to meet the goals of the study.

The decision to circumvent drug application was motivated by ethical and practical considerations. By pre-spiking the blood collection tubes with the enalapril/enalaprilat solution, considerations regarding inter-individual differences in enalapril absorption and metabolism could be disregarded. This approach additionally allowed for a less time consuming conduct of the feasibility study on-site. As no additional time was required to allow for drug absorption and metabolism after an oral application, sampling procedures could start right away. Moreover, this approach highly simplified processes to obtain ethical approvals, as it reduced the risk to which volunteers were exposed without limiting the objectives of the feasibility study.

During development of the study design, two different approaches for the evaluation of accuracy were pondered. On the one side an approache by establishing a reference group, in which blood collection and preparation happen under “ideal” conditions and on the other side a leaner approach by assessing the accuracy of PK/PD sampling/preparation by comparing the final concentration to the expected nominal concentration in the filled tube only. Using the reference group – approach in a comparable group size and composition as the verum group, allows to account for unexpected confounder of the pre-spiked approach effecting the accuracy of the final concentration in the blood collection tubes. Thus, the reference group approach appeared best to control the extent of unexpected/non-investigated effects (e.g. absorption of drug to plastic, degradation during storage, mistake in preparation of spiking solution etc.).

The substantial impact of the haematocrit concerning the established range of overall acceptable accuracy (58–153%) is caused by the use of reference values as individual haematocrit values were not determine within this investigation. Without the possibility of individual adjustment of analytical results, the whole range of normal haematocrits for participants of both genders had to be considered. A further stratification of results into a female and a male subgroup due to different haematocrit ranges was omitted based on the small number of participants per group, though this approach would have allowed the application of slightly stricter acceptance ranges by considering sex-specific haematocrit ranges (66.2–141.8% [overall range for male] and 65.5–145.5% [overall range for female], respectively). However, there would be no effect on the overall study outcome by using the sex-specific ranges if compared to the combined approach.

Corresponding to the authors' expectations, the pharmacodynamic humoral parameters investigated were found to be above given physiological lower reference values and within normal ranges for most of the volunteers. Some samples exhibited elevated values of renin and plasma renin activity. RAA system-related substances are known to be influenced by, for example, circadian rhythm, physical activity and posture during sampling. The elevated results were attributed to the study design, which did not dictate a specific time of day for sampling nor a preceding resting period.

Applicability of the here presented concept to other studies depends on the respective substances of interest. With regard to tested peptides/hormones, their presence in healthy adults would determine whether this concept for a feasibility study is applicable. If these criteria are met, such a feasibility study can aid a main trials' start-up process with regard to training of clinical staff and help evaluate as well as optimize planned procedures for studies with complex and demanding sampling procedures.

## 5 Conclusion

The feasibility study enabled investigation of each clinical team's ability to conduct protocol-conforming complex sampling and sample preparation procedures, sample logistics and bioanalysis. It facilitated determination of bottlenecks and optimised processes without endangering the vulnerable paediatric patients and avoiding low-quality data in the pivotal study. For studies with complex sampling and sample preparation procedures of non-standard parameters, which exceed clinical routine, the conduct of such a feasibility study proved to be a promising tool for verifying an appropriate trial conduct upfront.

## Author contributions

B.B.B. and A.M.C. developed the concept and protocol of this study. They conducted the investigation and analysed the samples supported by a bioanalytical team. Data interpretation was performed by A.M.C. supported by B.B.B. and S.L. All authors discussed the results. The manuscript was written by A.M.C and B.B.B. The final manuscript was critically reviewed by S.L.

## Declaration of interest

The authors declare that they have no conflict of interest.

## Funding

The research leading to these results has received funding from the European Union Seventh Framework Programme (FP7/2007–2013) under grant agreement n°602295 (LENA).
